# Linking scores on the 4- and 5-item versions of the Satisfaction with Life Scale in people with traumatic brain, spinal cord, or burn injury: a National Institute on Disability, Independent Living, and Rehabilitation Research Model System study

**DOI:** 10.1186/s41687-021-00335-9

**Published:** 2021-07-17

**Authors:** Alyssa M. Bamer, Kara McMullen, Anne Deutsch, Mitch Sevigny, Tracy Mroz, Shelly A. Wiechman, Jeffrey C. Schneider, Dagmar Amtmann

**Affiliations:** 1 Department of Rehabilitation Medicine, University of Washington, University of Washington Center on Outcomes Research in Rehabilitation, 12360 Lake City Way, Suite 502, Seattle, WA 98125 USA; 2 Shirley Ryan AbilityLab and Northwestern University, 355 E Erie St, Chicago, IL 60611 USA; 3 RTI International, 230 W Monroe St, Chicago, IL 60606 USA; 4 Spinal Cord Injury Model System, Craig Hospital, 3425 S. Clarkson Street, Englewood, CO 80113 USA; 5 Department of Rehabilitation Medicine, University of Washington, 1959 NE Pacific Street, Box 356490, Seattle, WA 98195 USA; 6 Department of Rehabilitation Medicine, University of Washington, 325 Ninth Ave, Box 359612, Seattle, WA 98104 USA; 7 Department of Physical Medicine and Rehabilitation, Harvard Medical School, Spaulding Research Institute, Spaulding Rehabilitation Hospital, 300 1st Avenue, Boston, MA 02129 USA

## Abstract

**Background:**

The Satisfaction with Life Scale (SWLS) is a widely used measure of subjective well-being. Recent evidence indicates the fifth item of the scale reduces the reliability of the scale and is inappropriate for use in traumatic injury populations. The purpose of this study was to develop a linking procedure between the five-item version of the SWLS and a modified four-item version, which removes the problematic item, for use in Spinal Cord (SCI), Traumatic Brain (TBI), and Burn Injury populations.

**Methods:**

Proration (i.e. adding the mean of the four items to their total) was identified as a potential linking solution that could be easily implemented in clinical or research settings. The validity of the proration approach was evaluated by examining mean differences, cross group classification by SWLS category, score correlations, the intraclass correlation coefficient, and visual inspection of Bland-Altman plots in a large sample of SCI, TBI, and Burn Injury survivors who were participants in the National Institute on Disability, Independent Living, and Rehabilitation Research (NIDILRR) Model Systems’ National Databases.

**Results:**

A total of 17,897 (SCI *n* = 8566, TBI *n* = 7941, and Burn *n* = 1390) participants were included in this study. SWLS scores ranged from 5 to 35, and the average score difference between directly derived and prorated scores was 0.39 points. A large majority of the sample (93%) had score differences of < 4 points (i.e. approximately 0.5 SD). The correlation between the prorated and directly derived scores was very high (*r =* 0.97) and the ICC value indicated excellent reliability (ICC = 0.97).

**Conclusions:**

This study provides a valid scoring approach for researchers or clinicians who don’t want to lose continuity with previously collected data but prefer to switch to the modified four-item version of the SWLS. Clear guidance is provided for traumatic injury researchers or clinicians on how to implement the proration scoring approach.

## Introduction

Satisfaction with life is a global construct of subjective well-being that has been widely studied across populations and fields, from economics to health and environment. The most commonly used measure of satisfaction with life is the Satisfaction With Life Scale (SWLS) [1], with extensive worldwide translations and administrations since its development in 1985. Though a significant number of studies have provided validity evidence for the SWLS, a review of the scale by Pavot and Diener [2] acknowledged that the fifth item of the scale, “If I could live my life over, I would change almost nothing,” has consistently lower factor loadings. They hypothesized that this is because the fifth item is an indirect indicator of satisfaction with life while the first four items are direct indicators (see Table 1 for all items). In addition, a recent psychometric evaluation of the SWLS by Amtmann et al. [3] in a large sample of traumatic injury (spinal cord (SCI), traumatic brain (TBI), and burn) populations found that the fifth item functions poorly and reduces the reliability of the scale. Based on this, and the fact that some individuals who have suffered a traumatic injury find the fifth item offensive [4], Amtmann et al. recommended dropping the fifth item from scale when used in traumatic injury populations.

**Table 1:** Satisfaction with life scale items and response set

Item number	Item text	Response set
Item 1	In most ways my life is close to my ideal.	7 = Strongly agree 6 = Agree 5 = Slightly agree 4 = Neither agree nor disagree 3 = Slightly disagree 2 = Disagree 1 = Strongly disagree
Item 2	The conditions of my life are excellent.	
Item 3	I am satisfied with my life.	
Item 4	So far I have gotten the important things I want in life.	
Item 5	If I could live my life over, I would change almost nothing.	

While dropping the fifth item may be appealing to improve scale functioning and address concerns about its appropriateness in traumatic injury populations, this solution has drawbacks. The extensive adoption of the SWLS worldwide means that any changes to the SWLS could result in a lost ability to directly compare the score based on the modified scale with past studies and those done in other populations that report a score based on all five items. One method to address this concern is to develop a crosswalk or linking table [5]. A linking table allows researchers to convert and compare scores on the new modified four-item scale with those on the original five-item scale.

A variety of linking methods exist including prediction, scale alignment, and equating [5]. In this study, we primarily considered “proration” as a linking solution. By “proration” we mean creating a prorated scale score by adding the average of the four items’ responses to their summary score or total. In other words, if only the four-item scale is administered, the fifth item is treated as missing data and imputed using the mean of the observed four item scores. This approach is often employed across the literature by researchers dealing with small amounts of item level missing data within a scale [6]. Graham [7] suggested that this method is reasonable when the following conditions are met: (1) a high proportion of the items in a scale can be used to generate the missing score, (2) item-total correlations are similar, and (3) internal consistency of the scale is high. Based on the results published by Amtmann et al. [3], all three of these conditions are met by the SWLS. The simplicity of proration also means that it can be easily implemented by clinicians and researchers who wish to utilize the four-item SWLS while maintaining the ability to convert scores to the five-item scale metric. This approach also results in all prorated or imputed scores falling within the actual range of the scale, and does not rely on the assumption of a normal distribution of test scores.

Therefore, the purpose of this study was to determine if proration could be used to link scores between the modified and original SWLS in traumatic injury populations so that clinicians and researchers can more easily administer a four-item version of the SWLS to improve psychometric properties and acceptability of the scale in traumatic injury populations.

## Methods

### Participants

The same dataset was used for this study as was used to conduct the psychometric evaluation of the SWLS by Amtmann et al. [3], and the study sample is described in more detail in the previous manuscript. In brief, this study included individuals enrolled in the SCI [8], TBI [9], and Burn [10] Model Systems. Only participants with complete data on the SWLS were included in the study. SWLS data were collected using a variety of methods across the model systems including in-person interview, phone interview, or mailed questionaries. Data utilized in this study were collected at one-year post-injury between 1989 (TBI), 1993 (Burn), or 1995 (SCI) and 2014 (SCI, TBI) or 2015 (Burn). Current inclusion criteria for the three model systems are described in detail elsewhere [11–13]. All participants provided informed consent according to institutional review board approved procedures at each Model System center.

### Measures

The SWLS assesses global quality of life and satisfaction using five items [1] (see Table 1). Total scores are generated by summing the five item scores, with total scores ranging from 5 to 35. Higher scores indicate greater life satisfaction. The following cutoffs can be used as benchmarks for interpreting scores: 31–35 *extremely satisfied*, 26–30 *satisfied*, 20–25 *neutral or slightly satisfied*, 15–19 *slightly dissatisfied*, 10–14 *dissatisfied*, and 5–9 *extremely dissatisfied* [14].

### Analyses

As a first step we generated prorated SWLS scale scores by averaging the first four items and adding that average score to the sum of the four items. As discussed above, we chose the proration approach due to its simplicity and because the original scale uses a simple summed score. Subsequent analyses refer to the directly derived SWLS score (i.e. the 5 item full scale score) and the prorated score. Normality of the scores was examined using histograms to verify subsequent statistical analyses were appropriate.

Prorated and directly derived scores were compared in the overall sample and within the three injury groups separately. We examined means of score differences and absolute values of score differences, visual inspection of Bland-Altman plots, cross-group classification by cutoff scores, score correlations, and the intraclass correlation coefficient (ICC). Bland-Altman plots [15] were constructed to visually present the difference between the actual and the prorated scores within each injury group. The Bland-Altman 95% limits of agreement for the overall sample were also calculated (mean difference ± 1.96 × Standard Deviation (SD) of the difference). We examined cross-group classification by determining how many participants switched into a different satisfaction category based on the cutoff benchmarks recommended by the scale author and described previously [14]. A Pearson’s correlation was calculated for the directly derived and the prorated score, with values greater than 0.9 considered very high positive correlation [16]. We computed ICC two-way mixed effects models with absolute agreement (type 3,1) to evaluate the correspondence between the direct and prorated scores. ICC values were interpreted as poor (< 0.5), moderate (0.5–0.75), good (0.75–0.9), and excellent (> 0.9) respectively based on recommended guidelines [17].

## Results

### Participants

A total of 17,897 (SCI *n* = 8566, TBI *n* = 7941, and Burn *n* = 1390) participants were included in this study. The majority of the sample was white (69%), male (75%), and the average age of the sample was 39 years (SD 17.6). Additional characteristics of the study sample can be found in Amtmann et al. [3].

### Validity analyses

**Fig. 1:**
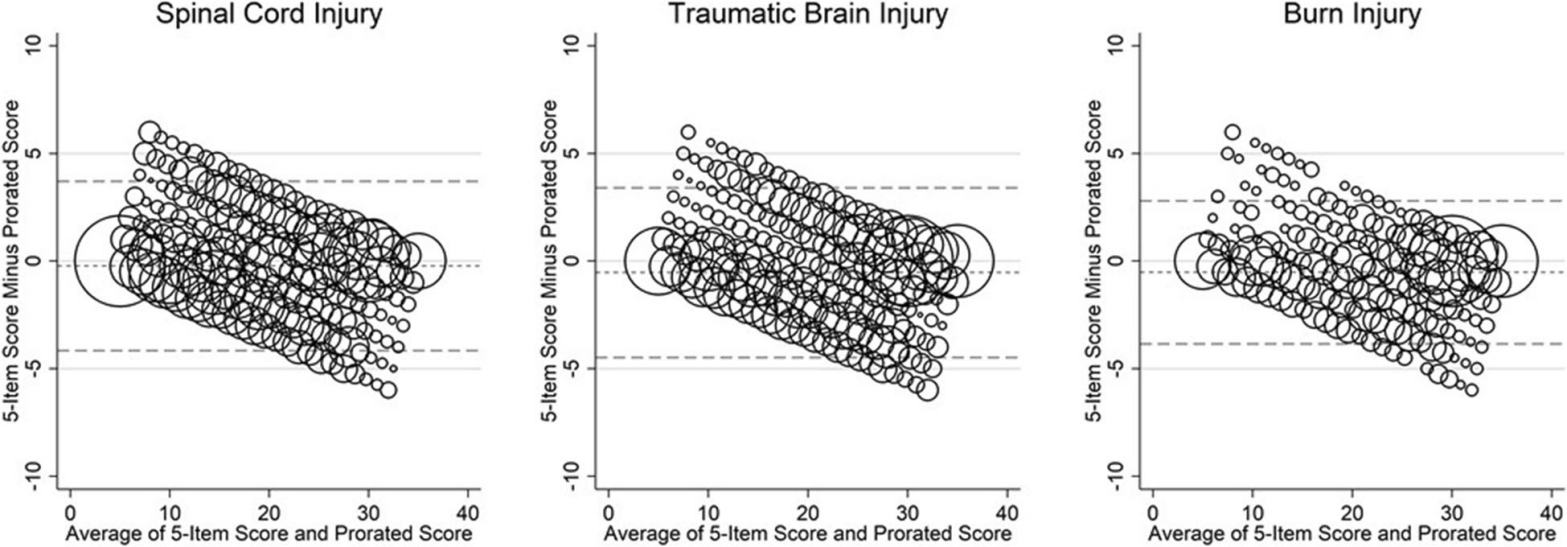
Bland-Altman plots for agreement between directly derived and prorated SWLS scores within each injury group. The small dotted line represents the mean difference between the scores and the large dashed lines represent the 95% limits of agreement

In the overall sample the average SWLS item level means were 3.76 (SD:2.07), 3.80 (SD:2.04), 4.32 (SD:2.07), 4.53 (SD:2.00), and 3.72 (2.18) for items 1 to 5, respectively. Because the mean for item 5 was lower than the other four items, the average score difference calculated by subtracting the prorated score from the directly derived score was slightly negative at − 0.39 (i.e. the prorated score is slightly larger on average). Within injury groups, mean differences were slightly smaller in SCI than in TBI or Burn (see Table 2). The Bland-Altman plots demonstrate that at low SWL values, the average differences are more positive (i.e. the 5-item direct score is higher) while at high SWLS scores the differences are generally negative (i.e. the prorated score is higher) (see Fig. 1). The 95% limits of agreement for the overall sample are − 4.3 to 3.5, and are − 4.2 to 3.7 for SCI, − 4.5 to 3.4 for TBI, and − 3.8 to 2.8 for Burn. The percentage of the sample with mean differences < 2 points is 67%, < 3 points is 83%, and < 4 points (i.e. approximately 0.5 SD) is 93%. Similarly, when classified into SWL category, 71% of the sample would be classified into the same category using either score. No individuals moved by more than one category up or down, with 8% classified into a lower SWL group and 20% into a higher SWL category using the prorated score compared to the directly derived score. Correlations between the prorated and directly derived scores were very high for the overall sample and within each injury group (all ≥0.97). Similarly, all ICC values displayed excellent reliability (all ICC ≥0.97) (see Table 2 for detailed results).

**Table 2:** Agreement between directly derived and prorated scores of the satisfaction with life scale in the overall sample and within TBI, SCI, and burn injury subsamples

		4-Item SWL Score (a)	Direct 5-Item SWL Score (b)	Prorated 5-Item SWL Score (c)	Score Difference (b-c)	Absolute value of difference (|b-c|)	Correlation (b with c)	ICC (3,1) (b with c)
	n	Mean (SD)	Mean (SD)	Mean (SD)	Mean (SD)	Mean (SD)		ICC (95% CI)
Overall Sample	17,897	16.4 (6.9)	20.1 (8.2)	20.5 (8.6)	−0.39 (1.95)	1.46 (1.35)	0.97	0.97 (0.97–0.97)
Spinal Cord Injury	8566	15.2 (6.7)	18.8 (7.9)	19.0 (8.3)	−0.23 (1.96)	1.45 (1.35)	0.97	0.97 (0.97–0.97)
Traumatic Brain Injury	7941	17.4 (6.9)	21.2 (8.2)	21.7 (8.6)	−0.54 (1.97)	1.52 (1.37)	0.97	0.97 (0.97–0.97)
Burn Injury	1390	18.2 (7.0)	22.2 (8.5)	22.8 (8.7)	−0.53 (1.66)	1.20 (1.26)	0.98	0.98 (0.98–0.98)

## Discussion

**Fig. 2:**
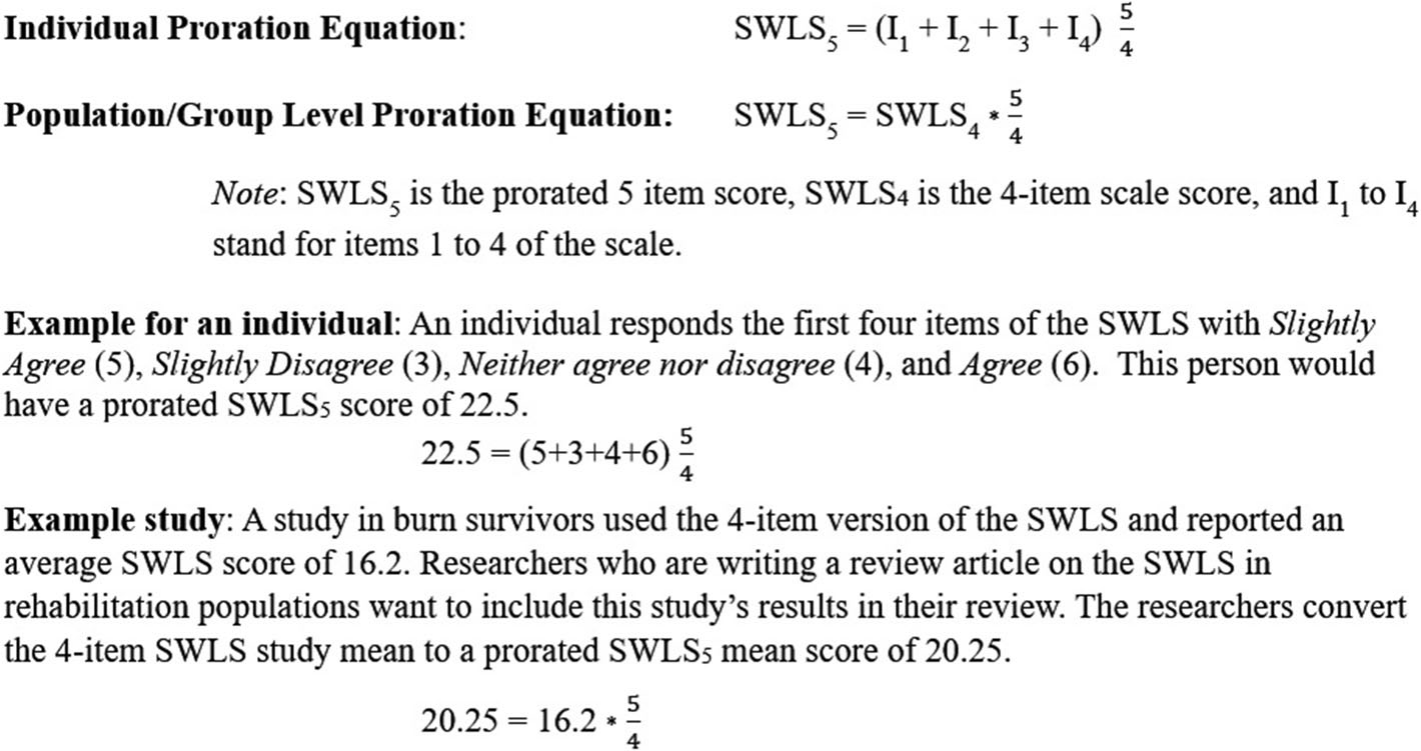
Equations and examples of how to implement the recommended proration technique at both the individual and group level

This study has demonstrated that by using a simple proration approach it is possible to generate a reliable estimate of a full SWLS score using the first four items of the scale. In addition, generating a prorated score only requires the use of a simple mathematical formula, and can be implemented at both the individual person level as well as for the mean of a group. Figure 2 provides step by step calculations to estimate a SWLS score based on all five items from the first four items and provides two examples for how to implement the proration approach.

The ability to link scores on the four-item scale with the five-item scale is important as the fifth item in the commonly used SWLS reduces the reliability of the scale [3] and is insensitive to the experiences of the trauma population [4]. In addition, feedback from Model Systems data collectors indicates some participants in the Model Systems studies find the fifth item difficult to answer. The fifth item, which asks responders if they would change almost nothing about their life, has the lowest item level mean of the five items in the scale. Thus, the overall prorated mean is slightly larger than the directly estimated mean, though this difference is less than half a point, which is only 5% of one standard deviation.

Translating the score does introduce additional noise into the cross-walked scores, as is true for any method of score transformation. Mean imputation can reduce variability in the data, underestimate standard deviation and variance and increased alpha [6]. However, in this study the standard deviation actually increased using the prorated score. Other alternatives, such as multiple imputation or full information maximum likelihood estimation, could also be used to estimate individual scores after administration of the proposed four-item scale. These have the benefit of reducing bias that can result from proration [6]. However, these approaches have a significant drawback in that they require complex software and can not easily be implemented by institutions with limited analytical resources or in clinical settings. In addition, if only the four-item scale is administered, the fifth item is no longer missing at random and the ideal solution is to utilize the four-item score in place of a five-item score whenever possible. Future studies should also examine the acceptability and reliability of the fifth item in other non-trauma populations, as other clinical populations may also find the item offensive or difficult to answer, and the four-item scale may prove to be more applicable across multiple populations.

One limitation of this study is that it included only individuals with moderate to severe traumatic injuries treated in Model System centers. While it is likely that the same proration approach could successfully be implemented in people with mild injuries or people with other health conditions, further evaluation should be completed before applying the approach more broadly. In addition, clinicians who utilize the modified four-item SWLS with a prorated score need to be aware that individual differences of four points or less could be due to random error or attributed to linking, though the majority of the sample had score differences of less than 2 points between the prorated and directly estimated scores.

In conclusion, a linking approach is necessary in order to compare scores across samples or studies when only a five-item summary score is available. However, when item level data is available, the preferred approach would be to compare four-item SWLS scores directly rather than impute the 5-item score due to introduction of bias. In situations where a 5-item score is required, the proration approach used in this study enables scores from the four-item version of the SWLS to be linked to the five-item version, and can be implemented both at the individual and group level clinically and in research settings.

## Data Availability

The datasets analyzed during the current study are available from burndata@uw.edu on reasonable request. In addition, all three Model Systems provide free public access to their data. See these websites for more details: https://www.nscisc.uab.edu, https://www.tbindsc.org/, and https://burndata.washington.edu/.

## Abbreviations

SWLS: Satisfaction with Life Scale
SCI: Spinal cord injury
TBI: Traumatic brain injury
ICC: intraclass correlation coefficient
SD: Standard Deviation
